# The Role of Irradiation in the Management of Locally Recurrent Non-Metastatic Soft Tissue Sarcoma of Extremity/Trunkal Locations

**DOI:** 10.1080/13577140412331332785

**Published:** 2004

**Authors:** James Fontanesi, Michael P. Mott, David R. Lucas, Peter R. Miller, Michael J. Kraut

**Affiliations:** 1 Department of Radiation Oncology Barbara Ann Karmanos Cancer Institute Wayne State University School of Medicine Detroit MI USA; 2 Department of Orthopaedic Surgery Barbara Ann Karmanos Cancer Institute Wayne State University School of Medicine Detroit MI USA; 3 Department of Pathology Barbara Ann Karmanos Cancer Institute Wayne State University School of Medicine Detroit MI USA; 4 Department of Medicine Barbara Ann Karmanos Cancer Institute Wayne State University School of Medicine Detroit MI USA; 5 Department of Radiology Barbara Ann Karmanos Cancer Institute Wayne State University School of Medicine Detroit MI USA; 6 Department of Medicine University of Michigan School of Medicine Detroit MI USA; 7 Department of Radiation Oncology Cedars Sinai Medical Center 8700 Beverly Blvd. C-2000 Los Angeles CA 90048 USA

## Abstract

*Background:* Patients who have had initial curative intent therapy for non-metastatic soft tissue sarcoma, and who subsequently relapse at the initial site without evidence of metastatic disease, have various options regarding local treatment. The treatment options available will be determined by the extent of relapse, previous therapy rendered, and patient characteristics. We reported on a series of 31 patients treated initially with only surgery for extremity/trunkal high-grade soft tissue sarcoma and then seen for recurrence at our institution between 1980 and 1999. Local re-treatment consisted of combined modality therapy, most often aggressive surgical debulking/resection and irradiation, in an effort to reduce the need for amputation and, where anatomically allowable, to maintain a functional limb. We report our results in re-establishing local control, subsequent survival, and complication rates.

*Methods:* Thirty-one patients with locally recurrent, non-metastatic high-grade soft tissue sarcoma, (excluding extraabdominal desmoid) were retrospectively reviewed to determine local control, survival, and complication rates associated with the relapsed disease. All patients had multimodality re-treatment most often utilizing aggressive surgical debulking and irradiation. The irradiation consisted of either external beam alone, brachytherapy alone, or a combination of external beam and brachytherapy. Nine patients also received multi-agent, multi-cycle chemotherapy using various regimens. In addition, the impact of surgical margin at the time of re-resection (gross versus microscopic disease), radiation treatment type, total radiation dose delivered, size of relapse, histological sub-type, sex and age, were evaluated to determine if they had any impact on the re-establishment of local control and subsequent survival.

*Results:* Local control was re-established in 25 of 31 (80.6%) patients. Two additional patients with isolated local relapse after irradiation were salvaged with amputation and remain NED at last follow-up. With these patients a total of 27/31 (87%) are now with local control. At last follow-up, which ranged from 23 to 192 months, 23 of 31 (74%) remained alive. Of the eight patients who have died, four had evidence of local and distant failure. Two additional patients died of distant failure while the treated sites remained in local control and two patients, both NED, died of intracurrent processes. Follow-up for those patients who had re-established local control has ranged from 23 to 192 months (median=60.5 months). Time to local failure following re-treatment ranged between 3 and 72 months following re-treatment (median=12 months). Five patients had significant treatment related complications. Included are two patients in which amputation was required due to local recurrences. Two patients developed a soft tissue necrosis and one patient had a wound healing problem that resolved with conservative management. No statistical significance in the development of local control could be found based on surgical margin status, total dose of irradiation (greater or less than 60 Gy), size of recurrence (greater than 5 cm), histological sub-type, sex, or age (greater than 50 years). There was a trend for negative impact for those patients receiving only external beam irradiation.

*Conclusion:* Selective locally recurrent, non-metastatic soft tissue sarcoma of the extremity/trunkal regions should still be considered eligible for aggressive limb-sparing therapy. Our experience suggests that a majority of patients re-establish local control following aggressive surgical resection/debulking and irradiation and this appears to be durable in its nature. The role of chemotherapy in this group of patients remains investigational. In a surprising finding, one patient re-relapsed in the re-treatment site at 72 months, thus justifying continued strict surveillance not only in the primary site but also for subsequent metastatic disease.


*Sarcoma*, June/September 2004, *VOL. 8, NO. 2/3, 57–61*

Taylor&Francis healthsciences

ORIGINAL ARTICLE

### The role of irradiation in the management of locally recurrent non-metastatic soft tissue sarcoma of extremity/trunkal locations

JAMES FONTANESI[1], MICHAEL P. MOTT[2], DAVID R. LUCAS[3], PETER R. MILLER[5] & MICHAEL J. KRAUT[4]

*Departments of [1]Radiation Oncology, [2]Orthopaedic Surgery, [3]Pathology, [4]Medicine, and [5]Radiology, Barbara Ann Karmanos Cancer Institute, Wayne State University School of Medicine, Detroit, MI, USA, & [5]Department of Medicine, University of Michigan School of Medicine, Detroit, MI, USA*

**Abstract**

*Background:* Patients who have had initial curative intent therapy for non-metastatic soft tissue sarcoma, and who subsequently relapse at the initial site without evidence of metastatic disease, have various options regarding local treatment. The treatment options available will be determined by the extent of relapse, previous therapy rendered, and patient characteristics. We reported on a series of 31 patients treated initially with only surgery for extremity/trunkal high-grade soft tissue sarcoma and then seen for recurrence at our institution between 1980 and 1999. Local re-treatment consisted of combined modality therapy, most often aggressive surgical debulking/resection and irradiation, in an effort to reduce the need for amputation and, where anatomically allowable, to maintain a functional limb. We report our results in re-establishing local control, subsequent survival, and complication rates.

*Methods:* Thirty-one patients with locally recurrent, non-metastatic high-grade soft tissue sarcoma, (excluding extra-abdominal desmoid) were retrospectively reviewed to determine local control, survival, and complication rates associated with the relapsed disease. All patients had multimodality re-treatment most often utilizing aggressive surgical debulking and irradiation. The irradiation consisted of either external beam alone, brachytherapy alone, or a combination of external beam and brachytherapy. Nine patients also received multi-agent, multi-cycle chemotherapy using various regimens. In addition, the impact of surgical margin at the time of re-resection (gross versus microscopic disease), radiation treatment type, total radiation dose delivered, size of relapse, histological sub-type, sex and age, were evaluated to determine if they had any impact on the re-establishment of local control and subsequent survival.

*Results:* Local control was re-established in 25 of 31 (80.6%) patients. Two additional patients with isolated local relapse after irradiation were salvaged with amputation and remain NED at last follow-up. With these patients a total of 27/31 (87%) are now with local control. At last follow-up, which ranged from 23 to 192 months, 23 of 31 (74%) remained alive. Of the eight patients who have died, four had evidence of local and distant failure. Two additional patients died of distant failure while the treated sites remained in local control and two patients, both NED, died of intracurrent processes. Follow-up for those patients who had re-established local control has ranged from 23 to 192 months (median = 60.5 months). Time to local failure following re-treatment ranged between 3 and 72 months following re-treatment (median = 12 months). Five patients had significant treatment related complications. Included are two patients in which amputation was required due to local recurrences. Two patients developed a soft tissue necrosis and one patient had a wound healing problem that resolved with conservative management. No statistical significance in the development of local control could be found based on surgical margin status, total dose of irradiation (greater or less than 60 Gy), size of recurrence (greater than 5 cm), histological sub-type, sex, or age (greater than 50 years). There was a trend for negative impact for those patients receiving only external beam irradiation.

*Conclusion:* Selective locally recurrent, non-metastatic soft tissue sarcoma of the extremity/trunkal regions should still be considered eligible for aggressive limb-sparing therapy. Our experience suggests that a majority of patients re-establish local control following aggressive surgical resection/debulking and irradiation and this appears to be durable in its nature. The role of chemotherapy in this group of patients remains investigational. In a surprising finding, one patient re-relapsed in the re-treatment site at 72 months, thus justifying continued strict surveillance not only in the primary site but also for subsequent metastatic disease.

**Key words:** *sarcoma recurrence, radiotherapy, brachytherapy*

Correspondence to: James Fontanesi MD, Chairman, Department of Radiation Oncology, Cedars Sinai Medical Center, 8700 Beverly Blvd. C-2000, Los Angeles, CA 90048, USA. Tel.: +1–310–423–4204; Fax: +1–310–659–3332; E-mail: jfontane@csccc.com

1357-714X print/1369–1643 online © 2004 Taylor & Francis Ltd
DOI: 10.1080/13577140412331332785

### Introduction

The treatment of soft tissue sarcomas continues to undergo study as new combined modality strategies are designed to improve the ability to establish limb salvage and reduce the chance of metastatic spread.[1–4] While there has been impressive local control data using these strategies, there remains between 10 and 20% of patients in whom initial local control is not established.

The treatment options available to these patients are based not only on local disease issues, but whether or not metastatic disease is present. The number of patients in whom locally recurrent non-metastatic soft tissue sarcoma is present, has not been well-defined in the literature although it has been reported in up to half of those who locally relapse.[5,6]

In an era where 80–90% of local control rates are reported using combined modality therapy for initial treatment, these locally relapsed patients are unique in that they may benefit from aggressive local re-treatment and may enjoy long-term disease-free survival.

It has been reported that certain prognostic factors such as size (greater than 5 cm), status of surgical margin, histological sub-type, age, and total dose of irradiation may also impact on local control and survival.[7–10]

The objective of this retrospective review was to report on local control, overall survival and complication rates in those patients receiving re-treatment using combined modality therapy. Additionally, we reviewed the relationship of the status of surgical margin at the time of re-treatment, type of radiation used, total dose of radiation, size of relapse, histological sub-type, sex and age, to determine if any of these factors impacted on local control or overall survival.

### Methods and materials

A retrospective review of all patients seen in the Department of Radiation Oncology at the Karmanos Cancer Institute/Detroit Medical Center from 1980 to 1999, who presented with recurrent soft tissue sarcoma, was undertaken. One hundred and two patients were identified, of which 31 had biopsy-proven high grade, locally relapsed soft tissue sarcoma of an extremity/trunkal site without evidence of metastatic disease. Patient characteristics are listed in Table 1. There were 17 males and 14 females; ages ranged from 18 to 88 years at the time of initial diagnosis. Sites included lower extremity ($n = 17$), trunkal ($n = 10$), and upper extremity ($n = 4$).

The most frequently noted histology was liposarcoma ($n = 8$) followed by malignant fibrohistiocytoma (MFH; $n = 7$), leiomyosarcoma ($n = 6$), and synovial sarcoma ($n = 3$). Tumor characteristics are noted in Table 2.

**Table 1.** *Patient characteristics*

| | |
|---|---|
| Male | $n = 17$ |
| Female | $n = 14$ |
| Ages | 18–88 years<br>Median $n = 53$ years |
| Time to re-treatment | 2–190 months<br>Median = 36 months |
| Follow up | 23–192 months<br>Median = 60.5 months |
| Local control | 25/31 (80.6%) |
| Local failure time from re-treatment to local failure | 6/31 (19.4%): 3–72 months (Median = 12 months) |

**Table 2.** *Tumor characteristics*

| | | |
|---|---|---|
| | Liposarcoma | $n = 8$ |
| | MFH | $n = 7$ |
| | Leiomyosarcoma | $n = 6$ |
| | All others | $n = 10$ |
| Site | | |
| | Lower extremity | $n = 17$ |
| | Trunkal | $n = 10$ |
| | Upper extremity | $n = 4$ |
| Radiation treatment schema | | |
| | External beam | $n = 16$ |
| | Brachytherapy | $n = 4$ |
| | External beam and brachytherapy | $n = 11$ |
| Surgical Margins (last surgery) | | |
| | Microscopic positive or within 5 mm | $n = 25$ |
| | Grossly positive | $n = 6$ |
| Size of relapse | | |
| | $\geq 5$ cm | $n = 19$ |
| | $\leq 5$ cm | $n = 12$ |

All patients had a previous history of surgical extirpation. Time to initial relapse from initial surgery ranged from 2 to 190 months (median = 36 months). Twelve patients had a history of multiple resections for local relapse prior to treatment with combined modalities. At the time of last surgery, the surgical margins were microscopically positive or close (within 3 mm) in 25 patients and grossly positive in six patients. These were reviewed by one of the authors (D.R.L.).

Irradiation was used in all patients and included external beam radiation alone ($n = 16$; dose range 45–68 Gy), brachytherapy alone ($n = 4$; two patients received neutron brachytherapy utilizing californium-252, two patients received high-dose rate remote afterloading using iridium-192), and 11 patients who had a combination of external beam irradiation and brachytherapy.

**Table 3.** *Patients characteristics of local failure*

| Patient | Age | Sex | Size at relapse | Site | Histology | Time to initial relapse | Surgical margins | Time to relapse | Status |
|---|---|---|---|---|---|---|---|---|---|
| 1 | 54 | ♂ | | Trunkal | MFH | 12 | M | 12 | ↓ LF/DD |
| 2 | 30 | ♀ | | Upper extremity | Synovial sarcoma | 60 | M | 12 | ↓ LF/DD |
| 3 | 40 | ♂ | | Upper extremity | MFH | 30 | M | 12 | ↓ LF/ DD |
| 4 | 75 | ♂ | | Lower extremity | RMS | 96 | M | 8 | ↓ LF/DD |
| 5 | 67 | ♂ | | Upper extremity | Leiomyosarcoma | 12 | M | 3 | NED following surgical salvage |
| 6 | 60 | ♂ | | Lower extremity | Synovial sarcoma | 6 | M | 72 | NED following surgical salvage |

Prior to 1994 there was no consensus departmental policy on what type of treatment or total dose to be used in this set of patients.

Beginning in 1994, high dose rate brachytherapy (HDR) was used as a 'boost' prior to initiation of external beam irradiation in patients with relapsed soft tissue sarcoma. The planned brachytherapy dose was to be 350 cGy to a 0.5-cm margin around surgically placed clips for Ir-192 HDR. For those treated with Cf-252, a neutron-emitting brachytherapy source, twice daily doses of 100 NcGy were delivered to the same volume as the HDR-treated patients. In combined cases, the HDR brachytherapy was immediately followed by initiation of external beam irradiation. The plan for this external beam was to deliver 200 cGy per day to a total dose of 50 Gy. The treatment volume ranged between 3 and 5 cm around the surgically placed clips.

For those receiving only brachytherapy with Cf-252 the total dose was between 7 and 9 NGy, again delivered twice daily and was used for patients with small lesions with microscopically positive surgical margins. We used an RBE of 4.5 when calculating neutron dose.

No patient has been lost to follow-up. Follow-up has ranged from 23 to 192 months, with a median of 60.5 months. This follow-up was started from the time of completion of the irradiation during the re-treatment.

### Results

Local control in the relapsed area was established in 25 of 31 (80.6%) patients. Follow-up for these patients has ranged from 23 to 192 months (median = 60.5 months).

Six patients developed local failure in the re-treatment area. No patient treated with brachytherapy alone failed, while two of 11 (18%) with combined external beam and brachytherapy had local failure. Four of sixteen (25%) treated with external beam irradiation alone developed local failure. Recurrence occurred from 3 to 72 months following completion of irradiation. The median time from re-treatment to relapse was 12 months. One patient had local failure at 72 months. Two of six patients were salvaged and both remain NED following surgical amputation. Time to death ranged from 13 to 121 months post radiation.

No patient treated with gross residual disease has locally failed; however, two have died of distant disease. All local failures have occurred in those treated for microscopically positive/close margins. Tumor characteristics of those patients with local failure are noted in Table 3.

There was no significance identified using univariate analysis for status of margin at the time of last surgery, the size of relapse, histology, total dose of radiation used, sex or age. There was a slight trend for improved local control in those patients receiving brachytherapy as part of their irradiation.

### *Survival*

At the time of last follow-up, 23 of 31 (74%) patients remained alive. Twenty-one have been disease free since completion of radiation. Two have had surgical salvage following local failure and remain NED at last follow-up. Follow-up for these patients ranges from 23 to 192 months (median = 60 months).

Eight patients have died. Four have died with a combination of local and distant failure and two have died of distant failure without local failure. Two patients have died of intracurrent disease, each had local control and no evidence of metastatic disease at the time of their death at 3 and 20 months following completion of irradiation. Those with local/distant failure died between 13 and 121 months following completion of irradiation. Univariate nor multivariate analysis could establish significance for any of the study factors as an independent reason influencing survival.

### *Complications*

As expected, most patients developed acute tissue reactions following irradiation. However, five of 31 (16%) developed significant complications. Two patients developed soft tissue necrosis. In one patient, 121 months had elapsed between initial diagnosis and re-treatment. This patient received only external beam irradiation (60 Gy/30 fractions). The treatment area was involved in trauma that resulted in a non-healing wound that required surgical intervention following failure of conservative healing measures.

The second patient who developed soft tissue necrosis received surgery, external beam irradiation and brachytherapy along with multiagent, multicycle chemotherapy at the time of re-treatment. This upper extremity site developed a wound dehiscence that also had positive bacterial cultures. This eventually healed with conservative measures. One patient developed a wound dehiscence following combined brachytherapy/external beam irradiation. This healed with conservative management within 6 weeks of development.

Two patients had amputation following local relapse. These patients had significant pain and/or functional loss of the affected areas. Following surgery, there was noted significant pain improvement and both remain NED.

### Discussion

The treatment of localized non-metastatic soft tissue sarcoma continues to undergo refinements designed to maximize local control and improve the ability to maintain function where possible. With most modern series reporting 80–90% local control rates, 5-year survival rates continue to range from 55 to 65%. Thus, while local control is excellent with modern therapy, investigations on how to decrease distant failure and improve overall survival has been the focus of much activity.[11–13] Since many patients receive multi-modality therapy, for those few with local relapse without evidence of metastatic disease, treatment options may be limited. Previous irradiation, limb-salvage surgery and chemotherapy can all affect potential new therapy that may be used.

Those who present with localized disease and who subsequently relapsed locally without evidence clinically or radiographically of distant failure, make up a relatively small number of patients. In fact, it has been reported that about 50% of patients with high grade soft tissue sarcomas who locally relapsed, will eventually develop metastatic disease within 24 months.[21] During the past 20 years, surgical treatment preferences have changed from amputation to limb salvage in conjunction with multi-modality treatments. Thus, there are an ever-increasing number of patients who may fit this unique category.

There are few series that report on the outcome of patients with local non-metastatic relapsed soft tissue sarcoma. Lewis *et al.* and Stojadinovic *et al.* reported on the Memorial Sloan-Kettering data for non-metastatic locally relapsed soft tissue sarcomas and found no survival difference from those who were referred at primary disease presentation.[14,15] An important note to these two series was the fact that recurrences greater than 5 cm, high-grade tumors, positive re-resection margins, and specific histologies, namely leiomyosarcoma and fibrosarcoma, predicted for poorer survival rates. This is different from the presented series in which neither histology, surgical margin, nor size were predictors of local control or survival. In fact, no patient with gross residual surgical margins has failed locally in the present series. In addition, it must be noted that all tumors in the present series were high grade. It is also important to know that each patient had high grade recurrent soft tissue sarcoma within 5 mm of the surgical margin. Thus, the 80% local control rate is encouraging as is the 74% survival rate.

There has been little information on the re-treatment of patients who are radiation naive at the time of local relapse. There have been limited reports of local control and survival following re-irradiation either alone or in conjunction with limb-salvage procedures and/or amputation. A real question is if radiation is really required, especially in those cases who undergo amputation and/or limb-salvage procedures with wide margins of resection. A recent series by Baldini *et al.* suggests that following adequate initial surgical resection with margins of resection playing the key indicator (100% local control for margins greater than 1 cm compared to 87% for those less than or equal to 1 cm) irradiation may not be necessary. The series also reported that tumor grade, size, site, nor depth had negative impact on local control.[16] This concept has also been reported by others.[17]

Our report of local control of 80% is similar to many series detailing initial presentation and treatment. Statistical analyses of our series found a trend for increasing local failure in those patients receiving only external beam irradiation for close surgical margins, although numbers receiving brachytherapy alone or in conjunction with brachytherapy may be too small to be able to accurately predict this fact. It may be that in these patients with relapsed local disease, that the overall time which was required to deliver external beam irradiation, may play an important part in the eventual ability to maintain local control.

We are also encouraged by the low incidence of treatment-related complications although if we include the patients who required amputation for local failure as a complication, this is still an acceptable 16%.

This retrospective review is narrow in its focus: locally recurrent, radiation-naive and non-metastatic soft tissue sarcoma. Despite this focus, it is encouraging that these patients have a substantial capacity for long-term, disease-free intervals following aggressive multi-modality therapy. We have not had an unusual rate of patients developing metastatic disease as has been previously reported in patients with high grade soft tissue sarcoma at the time of initial presentation. We also could not demonstrate that surgical margin status, time to local relapse, radiation dose, nor size were negatively predictive for poor outcomes. The only trend for poor local control was with the use of external beam irradiation alone in those patients with close margins.

### Conclusion

Based on this experience, we continue to recommend the aggressive surgical resection followed by irradiation using brachytherapy alone (for lesions less than 5 cm in size) or in conjunction with external beam irradiation for larger lesions. The role of chemotherapy should be reserved for those patients in a protocol setting since its use has yet to be fully identified and defined.

### References

1. Weitz J, Antonescu CR, Brennan MF. Localized extremity soft tissue sarcoma: improved knowledge with unchanged survival over time. *J Clin Oncol* 2003; 21(14): 2719–25.
2. Benjamin RS. Evidence for using adjuvant chemotherapy as standard treatment of soft tissue sarcoma. *Semin Radiat Oncol* 1999; 9(4): 349–51.
3. Issels RD, Schlemmer M. Current trials and new aspects in soft tissue sarcoma of adults. *Cancer Chemother Pharmacol* 2002; 49 (Suppl 1): S4–8.
4. Alektiar KM, Velasco J, Zelefsky MJ, Woodruff JM, Lewis JJ, Brennan MF. Adjuvant radiotherapy for margin-positive high-grade soft tissue sarcoma of the extremity. *Int J Radiat Oncol Biol Phys* 2000; 48(4): 1051–8.
5. Gibbs FJ, Lee RJ, Driscoll DL, McGrath BE, Mindell ER, Kraybill WG. Clinical importance of late recurrence in soft-tissue sarcomas. *J Surg Oncol* 2000; 73(2): 81–6.
6. Janjan N, Crane C, Delclos M, Ballo M. Brachytherapy for locally recurrent soft tissue sarcoma. *Am J Clin Oncol* 2002; 25(1): 9–15.
7. O'Sullivan B, Pisters PW. Staging and prognostic factor evaluation in soft tissue sarcoma. *Surg Oncol Clin N Am* 2009; 12(2): 333–53.
8. Eilber FC, Rosen G, Nelson SD, Selch M, Dorey F, Eckardt J, Eilber FR. High-grade extremity soft tissue sarcomas: factors predictive of local recurrence and its effect on morbidity and mortality. *Ann Surg* 2003; 237(2): 218–26.
9. Youssef E, Fontanesi J, Mott M, Kraut M, Lucas D, Mekhael H, Ben-Josef E. Long-term outcome of combined modality therapy in retroperitoneal and deep-trunk soft-tissue sarcoma: analysis of prognostic factors. *Int J Radiat Oncol Biol Phys* 2002; 54(2): 514–9.
10. Stojadinovic A, Leung DH, Hoos A, Jaques DP, Lewis JJ, Brennan MF. Analysis of the prognostic significance of microscopic margins in 2,084 localized primary adult soft tissue sarcomas. *Ann Surg* 2002; 235(3): 424–34.
11. Henshaw RM, Priebat DA, Perry DJ, Shmookler BM, Malawer MM. Survival after induction chemotherapy and surgical resection for high-grade soft tissue sarcoma. Is radiation necessary? *Ann Surg Oncol* 2001; 8(6): 484–95.
12. Ottaiano A, De Chiara A, Fazioli F, De Rosa V, Ravo V, *et al.* Neoadjuvant chemotherapy for intermediate/high-grade soft tissue sarcomas: five-year results with epirubicin and ifosfamide. *Anticancer Res* 2002; 22(6B): 3555–9.
13. Petrioli R, Coratti A, Correale P, D'Aniello C, Grimaldi L, *et al.* Adjuvant epirubicin with or without Ifosfamide for adult soft-tissue sarcoma. *Am J Clin Oncol* 2002; 25(5): 468–73.
14. Stojadinovic A, Yeh A, Brennan MF. Completely resected recurrent soft tissue sarcoma: primary anatomic site governs outcomes. *J Am Coll Surg* 2002; 194(4): 436–47.
15. Lewis JJ, Leung D, Heslin M, Woodruff JM, Brennan MF. Association of local recurrence with subsequent survival in extremity soft tissue sarcoma. *J Clin Oncol* 1997; 15(2): 646–52.
16. Baldini EH, Goldberg J, Jenner C, Manola JB, Demetri GD, Fletcher CD, Singer S. Long-term outcomes after function-sparing surgery without radiotherapy for soft tissue sarcoma of the extremities and trunk. *J Clin Oncol* 1999; 17(1): 3252–9.
17. Khanfir K, Alzieu L, Terrier P, Le Pechouz C, Bonvalot S, Vanel D, Le Cesne A. Does adjuvant radiation therapy increase loco-regional control after optimal resection of soft tissue sarcoma of the extremities? *Eur J Cancer* 2003; 39(13): 1872–80.
